# Design of a Novel and Selective IRAK4 Inhibitor Using Topological Water Network Analysis and Molecular Modeling Approaches

**DOI:** 10.3390/molecules23123136

**Published:** 2018-11-29

**Authors:** Myeong Hwi Lee, Anand Balupuri, Ye-rim Jung, Sungwook Choi, Areum Lee, Young Sik Cho, Nam Sook Kang

**Affiliations:** 1 Graduate School of New Drug Discovery and Development, Chungnam National University, 99 Daehak-ro, Yuseong-gu, Daejeon 34134, Korea; mhlee89@cnu.ac.kr (M.H.L.); balupuri@cnu.ac.kr (A.B.); daniel1356@naver.com (Y.-r.J.); swchoi2010@cnu.ac.kr (S.C.); 2 College of Pharmacy, Keimyung University, 1095 Dalgubeol-daero, Dalseo-Gu, Daegu 42601, Korea; a-rum925@hanmail.net (A.L.); yscho123@kmgw.kmu.ac.kr (Y.S.C.)

**Keywords:** Topological water network, kinase selectivity, IRAK4, pharmacophore mapping, virtual screening, molecular docking, molecular dynamics simulation

## Abstract

Protein kinases are deeply involved in immune-related diseases and various cancers. They are a potential target for structure-based drug discovery, since the general structure and characteristics of kinase domains are relatively well-known. However, the ATP binding sites in protein kinases, which serve as target sites, are highly conserved, and thus it is difficult to develop selective kinase inhibitors. To resolve this problem, we performed molecular dynamics simulations on 26 kinases in the aqueous solution, and analyzed topological water networks (TWNs) in their ATP binding sites. Repositioning of a known kinase inhibitor in the ATP binding sites of kinases that exhibited a TWN similar to interleukin-1 receptor-associated kinase 4 (IRAK4) allowed us to identify a hit molecule. Another hit molecule was obtained from a commercial chemical library using pharmacophore-based virtual screening and molecular docking approaches. Pharmacophoric features of the hit molecules were hybridized to design a novel compound that inhibited IRAK4 at low nanomolar levels in the in vitro assay.

## 1. Introduction

Protein kinases are enzymes that catalyze the phosphorylation of proteins, where phosphate groups are transferred from phosphate-donating molecules to specific substrates. Protein phosphorylation regulates cell cycles, and abnormal phosphorylation can cause several diseases. Protein kinases are attractive targets in structure-based drug discovery, because the general structure and characteristics of kinase domains are well-known [1]. They are regarded as primary drug targets in the pharmaceutical industry, due to their involvement in immune-related diseases, as well as various cancers [2]. Although the structural characteristics of adenosine triphosphate (ATP) binding sites of protein kinases are widely known, the development of selective kinase inhibitors remains a major challenge, due to their highly conserved nature. Selectivity for a specific target is critical, as poor selectivity may result in adverse effects. In the present study, we analyzed the water networks in the ATP binding sites of several kinases to address this problem. Binding site water molecules play an important role in protein–ligand recognition. They are either displaced upon ligand binding, or they form water bridges to stabilize the complex. Understanding of the behavior of water molecules in the active site of a protein greatly improves the characterization of protein–ligand binding. Binding site water molecules are commonly neglected in structure-based ligand discovery. However, their consideration can greatly increase the efficiency of rational drug design approaches. Water molecules have received considerable attention over the last few years. A variety of methods, including Dehydron [3], WaterMap [4], and SDO (solvent dipole ordering) [5] have been reported and employed to investigate the binding site. Water molecules in the binding site of the proteins, especially network-forming water molecules, could explain the characteristics of the site, and could be useful for the design of inhibitors. Our research group has developed a computational method for analyzing the water networks in the binding site of a protein. In this method, water network analysis is focused on the three-, four-, five-, and six-membered water rings. We named this approach as topological water network (TWN) analysis. Comprehensive details of this method are discussed in our former paper [6]. Our previous works revealed that TWNs in the binding site can explain the potency and selectivity of kinase inhibitors [6,7]. Here, we employed TWN analysis to develop an inhibitor for interleukin-1 receptor-associated kinase 4 (IRAK4).

IRAK4 is a crucial component in signal transduction pathways mediated by interleukin-1 receptors (IL-1Rs) and Toll-like receptors (TLRs). It plays critical roles in initiating innate immune responses and impacts adaptive immunity as well. Upon ligand binding, TLRs and IL-1Rs recruit the adaptor protein myeloid differentiation primary response 88 (MyD88) which in turn recruits IRAK4 and activate its kinase function [8]. Structural characteristics of IRAK4 can be determined from various available apo- and complex form structures [9,10,11,12,13,14,15,16,17]. Overall, IRAK4 structure is similar to a general kinase structure. However, it lacks a back pocket in the ATP binding site, due to a unique tyrosine gatekeeper residue, and its solvent-exposed region is more expanded than that of other kinases [9,10]. Recently, drugs targeting IRAK4 have been reported for the treatment of various immune diseases and cancers. Pfizer compound PF-06650833 is the first compound to complete Phase I clinical trials as an IRAK4 inhibitor (ClinicalTrials.gov Identifiers: NCT02485769, NCT02224651 and NCT02609139) and it is currently in clinical Phase II trials for rheumatoid arthritis (RA) (ClinicalTrials.gov Identifier: NCT02996500). Furthermore, it is also being studied in the treatment of cancers that specifically occur in the activated B cell subtype of diffuse large B cell lymphoma (ABC-DLBCL) patient populations with the *MyD88* L265P mutation [18,19].

**Figure 1:**
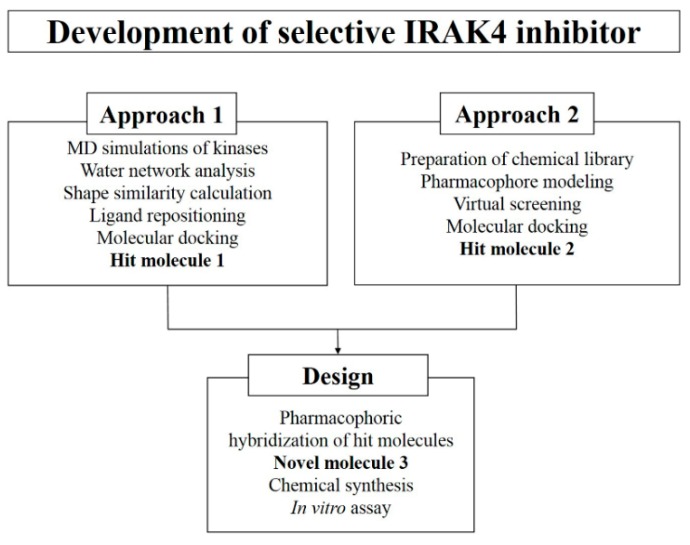
Flow chart illustrating the computer-aided design and development of a potent and selective IRAK4 inhibitor. Hit and designed molecules identified using different approaches are highlighted.

In the present study, we performed molecular dynamics (MD) simulations on IRAK4 and 25 other kinases (Table S1 of Supplementary Materials). Reasonably stabilized root mean square deviations (RMSD) curves for the simulated proteins during 2 ns MD simulation (Figure S1 of Supplementary Materials) suggested that they are suitable for further analysis. Subsequently, TWN propensity in their ATP binding sites (Table S2 of Supplementary Materials) were compared. Afterwards, we repositioned a known kinase inhibitor in the binding site of kinases which exhibited similar TWNs to obtain a hit molecule. Additionally, we performed pharmacophore-based virtual screening and molecular docking studies on a commercial chemical library to acquire one more hit molecule. Finally, we designed a novel compound on the basis of the pharmacophoric features of the hit molecules, and tested its inhibitory activity against IRAK4. A schematic overview of the design process is provided in Figure 1. 

## 2. Results and Discussion

### 2.1. TWN Analysis and Staurosporine-Based Repositioning

**Figure 2:**
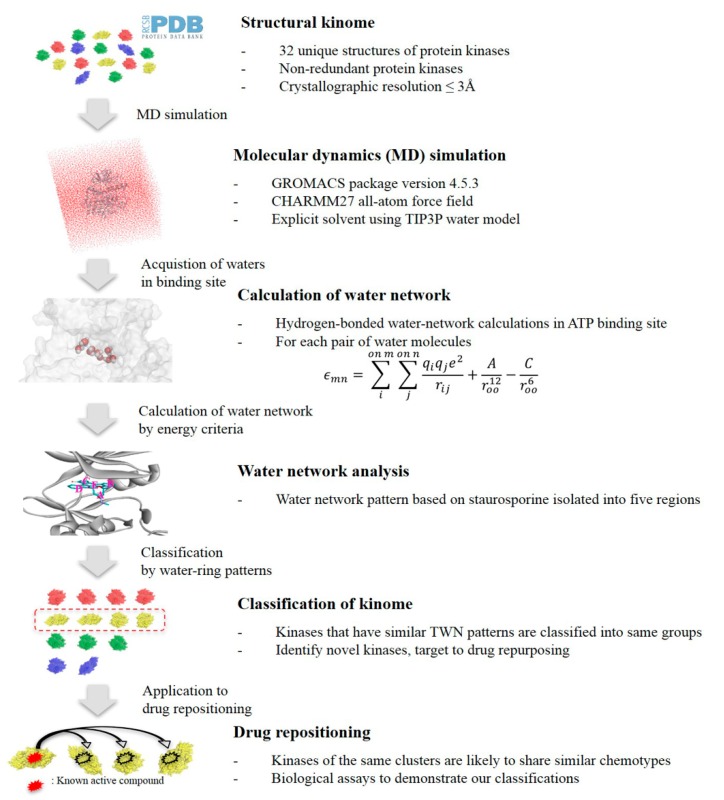
Flow chart describing the drug repositioning approach on the basis of topological water network (TWN) analysis.

**Figure 3:**
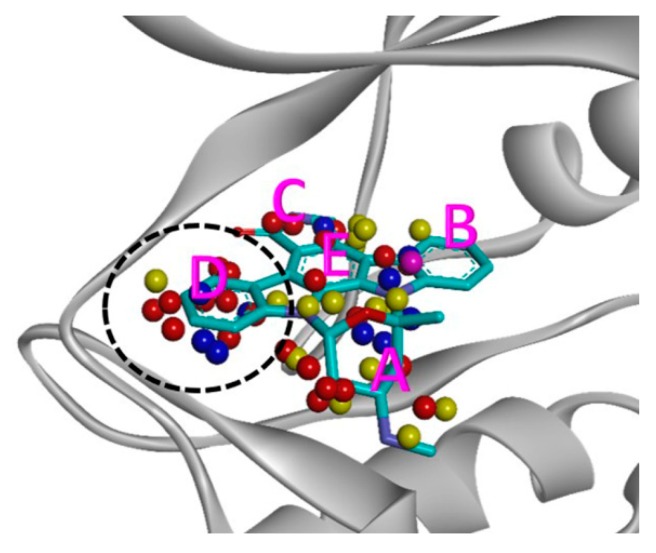
Superimposed TWNs around staurosporine in the IRAK4 binding pocket. We divided the ATP binding site into five regions (A–E) based on staurosporine’s binding mode, to analyze TWN patterns. Three-, four-, five-, and six-membered water rings are represented by red, yellow, blue, and purple spheres, respectively. The D site with a unique water network pattern is highlighted using a black dashed circle.

As shown in Figure 2, TWN analysis based on the staurosporine’s binding mode was carried out to obtain information about the drug repositioning between kinases. Staurosporine acts as an inhibitor of multi-kinases, and biological data is known for about 250 kinases [20]. It occupies the ATP binding sites of kinases due to its planar structure with few rotatable bonds. Complexed structures of staurosporine with many kinases are known [20]. Here, 26 such kinase–staurosporine complexed structures were examined (Table S1 of Supplementary Materials). As described in the experimental section, we performed MD simulations and analyzed TWNs in the binding site of the selected kinases. We calculated the frequency of hydrogen-bonded cyclic water rings, such as three-, four-, five- and six-membered rings. We checked the distribution of TWN in five regions, namely A–E, as shown in Figure 3. TWN analysis revealed a high percentage of water molecules in the D-site of the IRAK4 (35.7%) binding pocket. Similar to IRAK4, three more kinases, namely apoptosis signal-regulating kinase 1 (ASK1, 33.3%), tyrosine-protein kinase Lyn (LYN, 31.3%), and interleukin-2-inducible T-cell kinase (ITK, 26.0%) exhibited comparable TWN tendency in the D-site. A summary of the water networks in each region of the four kinases is provided in Table 1.

**Figure 4:**
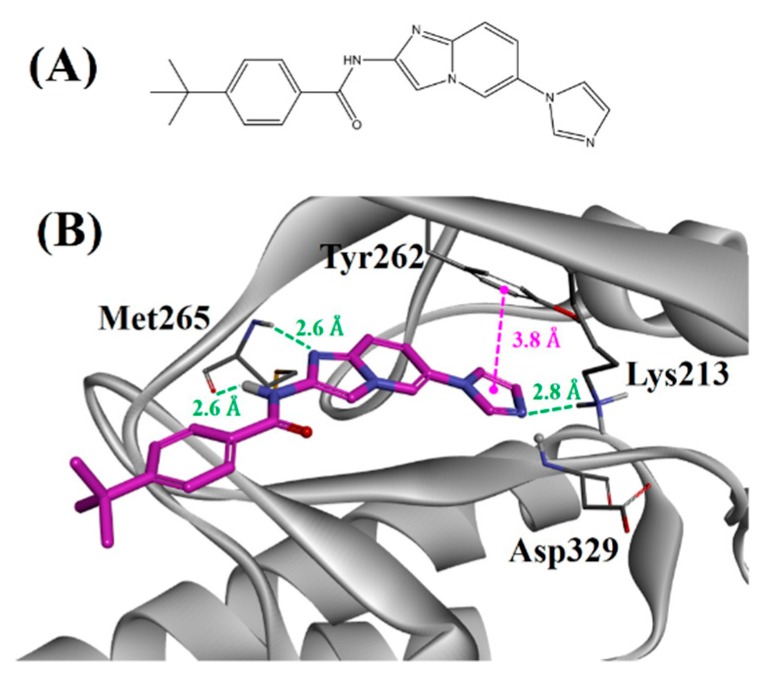
(**A**) Two-dimensional (2D) structure of compound **1**. (**B**) Binding mode of compound **1** against IRAK4. The green and magenta lines represent hydrogen bond and hydrophobic interactions, respectively.

We anticipated that a known kinase inhibitor could be repositioned by targeting the D-region of the kinases with a similar water network pattern. Compound **1** (Figure 4A) was released as an ASK1 inhibitor by the Takeda pharmaceutical company [21]. Furthermore, its binding mode with ASK1 is known, as the co-crystal structure is available [21]. Docking of compound **1** into the active site of IRAK4 (Figure 4B) showed that the ligand could cover the D-region, and its interactions with key residues “Lys213, Tyr262, and Met265” of the binding pocket suggested that it could inhibit IRAK4. Lys213, Tyr262, and Met265 are catalytic, gatekeepers and hinge residues of IRAK4, respectively. They are crucial for IRAK4 activity. Compound **1** showed two hydrogen bonds with hinge residues Met265 at a distance of 2.6 Å. In addition, it formed hydrogen bonds with the catalytic residue Lys213 at a distance of 2.8 Å. Tyr262 was involved in a hydrophobic interaction with the ligand at a distance of 3.8 Å.

Compound **1** was available through commercial vendors, and thus, we decided to verify its inhibitory activity against ASK1, IRAK4, LYN, and ITK through an in vitro assay. It showed significant inhibition against the original target ASK1 with an IC_50_ value of 53.0 nM. Additionally, IC_50_ values of 187.5 nM, 2385.5 nM, and 1670.5 nM were obtained against IRAK4, LYN, and ITK, respectively. As shown in Table 2, these kinases do not have high total sequence similarity and binding site similarity [22]. However, our TWN results revealed that drug repositioning is possible on the basis of the D-site. As shown in Table 1, IRAK4, ASK1, LYN, and ITK exhibited similar water network patterns in the D-region, with 35.7%, 33.3%, 31.3%, and 26.0% TWNs, respectively. In order to explore whether TWN similarity observed in the D-region is due to D-site sequence similarity, we calculated D-site sequence similarity and summarized this in Table S3 of Supplementary Materials. ASK1 and LYN showed 80% and 60% D-site sequence similarity, respectively, in comparison to IRAK4 (Table S3). TWN and D-site sequence similarity shows comparable patterns for IRAK4, ASK1, and LYN. However, a similar pattern was not observed for LYN and ITK, as both displayed 60% D-site sequence similarity. Likewise, a similar pattern was not observed for other kinases such as tyrosine-protein kinase Fes (FES) and LIM domain kinase 1 (LIMK1). As can be seen in Table S2 of supplementary materials, FES and LIMK1 showed almost identical water network patterns with 24.2% and 24.6% TWNs, respectively, in the D-site. In contrast, they exhibited 40% and 60% D-site sequence similarity, respectively (Table S3). These results suggest that drug repositioning is possible on the basis of the TWN pattern, irrespective of sequence similarity or dissimilarity. 

### 2.2. Selectivity Analysis by Shape Similarity

Besides confirming the possibility of drug repositioning using TWN analysis in the above process, we also calculated the shape similarity between the water molecules and the ligand to explain the selectivity. Shape similarity is the comparison between the ligand and the center of mass of water networks by using geometrical characteristics. For calculating the shape similarity, we docked compound **1** in the binding sites of IRAK4, ASK1, LYN and ITK. We acquired water networks whose centers of mass were located within 2 Å from compound **1** by using the in-house codes (Supplementary Material). Results for the shape similarity are summarized in Table 3. As discussed before, compound **1** exhibited higher inhibitory activity against ASK1 and IRAK4 than LYN and ITK. When compared using the entire ligand structure, comparable similarity was observed for ASK1 (24.2%) and IRAK4 (22.7%). Likewise, comparable similarity was observed for LYN (18.2%) and ITK (17.4%). The D-site, which proceeds to the solvent-exposed region, was found to be responsible for imparting this selectivity. Similarity around the D-site was found to be 60.7%, 67.6%, 49.2%, and 41.5% for ASK1, IRAK4, LYN, and ITK, respectively. The high potency of compound **1** against ASK1 (53.0 nM) and IRAK4 (187.5 nM) could be attributed to high D-site shape similarity (>50%). In the same way, a low potency of compound **1** against LYN (2385.5 nM) and ITK (1670.5 nM) could be attributed to low D-site shape similarity (<50%). Hence, differences in the inhibitory activity of compound **1** against the four kinases could be explained by dissimilar water environments in kinases and shape similarity. Our findings are in agreement with a previous study that reported the importance of the solvent-exposed region (D-region) of IRAK4 [23]. The inhibitory activity of a compound is influenced by the overall characteristics of the binding site of a specific protein kinase. Accordingly, TWN and shape similarity values in a particular site of a kinase cannot be precisely correlated with the inhibitory activity of a compound. This could be the reason for the comparatively lower activity of compound **1** against LYN (2385.5 nM) than ITK (1670.5 nM), even though LYN showed a relatively higher TWN (31.3%) and shape similarity (49.2%) than ITK (26.0% TWN and 41.5% shape similarity) in the D-site. Additionally, it can be seen in Table 1 that unlike IRAK4 and ASK1; LYN and ITK showed a higher percentage of water molecules in other regions than the D-site. TWN for LYN in the E-site was found to be 50%, while ITK exhibited 36% TWN in the A-site. 

### 2.3. Pharmacophore-Based Virtual Screening and Molecular Docking

**Figure 5:**
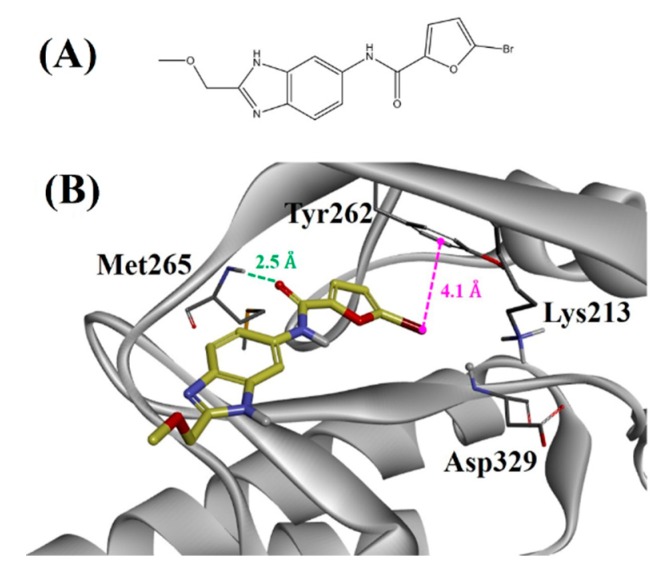
(**A**) Two-dimensional structure of compound **2**. (**B**) Binding mode of compound **2** against IRAK4. The green and magenta lines represent hydrogen bond and hydrophobic interactions, respectively.

We developed several pharmacophore models based on the HipHop algorithm. A model comprising of two hydrogen bond acceptors, one hydrogen bond donor, and two hydrophobic features, was carefully selected as the best pharmacophore model for virtual screening on the basis of rank score and cluster processes. This model was used as a query to search a library of 380 compounds that was prepared from lead-like library of OTAVA chemicals [24]. Compound **2** (Figure 5A) with a fit value of 1.9 was obtained as a hit molecule from the pharmacophore-based virtual screening. Furthermore, this molecule was docked inside the active site of IRAK4. Its predicted binding mode is displayed in Figure 5B. Compound **2** formed a hydrogen bond with the primary hinge residue Met265 at a distance of 2.5 Å. Additionally, its bromine atom showed a hydrophobic interaction with Tyr262 at a distance of 4.1 Å. Furthermore, we tested the inhibitory activity of compound **2** against IRAK4 using an in vitro assay where it exhibited an IC_50_ value of 6651.0 nM.

### 2.4. Design, Synthesis and Testing of IRAK4 Inhibitor 

**Figure 6:**
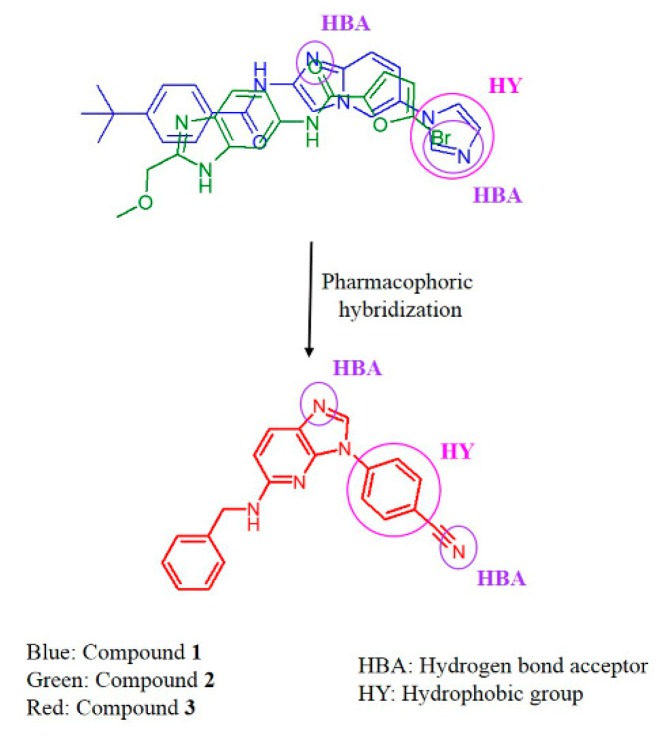
Design of a novel compound **3** from compounds **1** and **2**.

**Figure 7:**
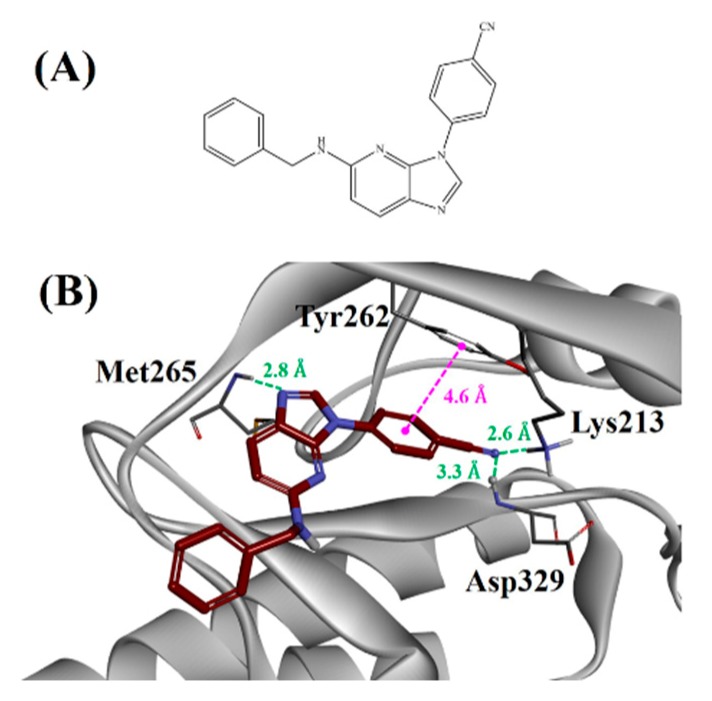
(**A**) Two-dimensional structure of compound **3**. (**B**) Binding mode of compound **3** against IRAK4. The green and magenta lines represent hydrogen bond and hydrophobic interactions, respectively.

Initially, two hit molecules (compounds **1** and **2**) were acquired using computational approaches such as TWN analysis, pharmacophore-based virtual screening, and molecular docking. As shown in Figure 6, pharmacophoric features of the hit molecules were hybridized to design a novel molecule (compound **3**). We designed a scaffold that maintains hydrogen bond interactions with catalytic residue, Lys213, along with hinge residue Met265 and hydrophobic interaction with the gatekeeper residue Tyr262. Figure 7A,B show the structure and predicted binding mode of compound **3**. It showed hydrogen bond interactions with Lys213, Met265, and Asp329 at distances of 2.6, 2.8, and 3.3 Å, respectively. Moreover, it exhibited a hydrophobic interaction with Tyr262 at a distance of 4.6 Å.

**Scheme 1:**
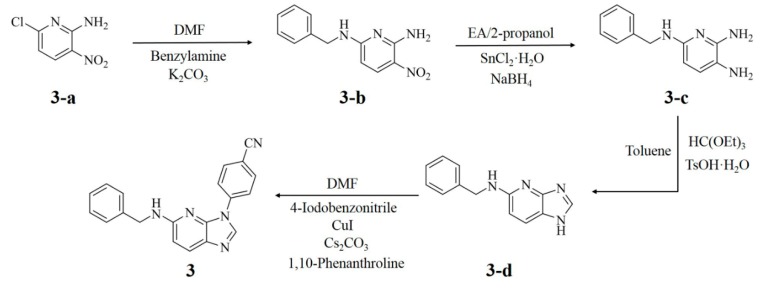
The synthetic route for compound **3**.

As compound **3** was designed through in silico approaches, we synthesized the molecule to examine its inhibitory activity against IRAK4. The synthesis process is outlined in Scheme 1. Commercially available compound **3**-**a** was reacted with a benzylamine in dimethylformamide (DMF) in the presence of potassium carbonate to obtain compound **3**-**b** (93% yield). Subsequently, the nitro group of compound **3**-**b** was reduced by using Tin(III) chloride and sodium borohydride to produce compound **3**-**c**. Cyclization of compound **3**-**c** in the presence of triethyl orthoformate provided compound **3**-**d**. Finally, compound **3** (34% yield) was prepared by the Ullmann reaction between 4-idobenzonitril and 4-azabenzimidazole (**3**-**d**) in the presence of copper(I) iodide, cesium carbonate, and 1,10-phenanthroline. Afterwards, an in vitro enzymatic assay was performed where compound **3** demonstrated 97% inhibition of IRAK4 at 10 μM. Its IC_50_ value was found to be 157.5 nM. Furthermore, compound **3** satisfies Lipinski’s ‘rule of five’ [25]. Properties such as molecular weight of 325.38, four hydrogen bond donors, one hydrogen bond acceptor, and a ClogP of 3.99 indicate its drug-like chemistry. Consequently, it is a suitable candidate for further investigation as an IRAK4 inhibitor. 

## 3. Materials and Methods

### 3.1. Protein Preparation

Crystal structures of various protein kinases were retrieved from the RCSB Protein Data Bank (PDB) [26]. All ligands and water molecules were removed from the original PDBs. Structures were examined for missing residues, and no such residues were found. Furthermore, bond orders and charges were inspected using the prepare protein protocol of Discovery Studio 2017 R2 (BIOVIA, San Diego, CA, USA). 

### 3.2. MD Simulation

MD simulations were performed using GROMACS 4.5.3 [27] and a CHARMM27 force field [28]. Protein was solvated in a cubic box of TIP3P water molecules [29] with a margin distance of 1.2 nm. Counter ions were added to neutralize the system. Energy minimization of the whole system was carried out for 500 steps using the steepest descent method. Subsequently, equilibration was performed in two phases. Phase 1 equilibration was conducted under an NVT ensemble (constant number of particles, volume, and temperature) to stabilize the temperature. NVT equilibration was carried out for 100 ps at 300 K using Berendsen thermostat [30]. Phase 2 was equilibration-conducted under an NPT ensemble (constant number of particles, pressure and temperature) to stabilize the pressure. NPT equilibration was performed for 200 ps with a Parrinello-Rahman barostat [31]. Finally, a production run for 2 ns with a time step of 1 fs was carried out at 300 K and a pressure of 1 bar. Periodic boundary conditions were applied in three dimensions. Coordinate trajectories were recorded every 20 ps for the MD run. Short-range electrostatic interactions were truncated at 1.2 nm, and long-range electrostatics were calculated using the Particle mesh Ewald (PME) method [32]. A linear constraint solver (LINCS) algorithm was employed to constrain all bond lengths [33].

### 3.3. TWN Analysis

Water molecules linked by hydrogen bonds form several types of hydrogen-bonded cyclic water-ring networks. Such networks are known as topological water networks (TWNs), and they include three-, four-, five- and six-membered rings. The potential functions considered in the TWNs involve a rigid water model, TIP3P. The interactions between water molecules are often modeled by the Lennard-Jones (LJ) and Coulomb interactions [29]. If v(a,b) is the interaction potential energy between water molecules a and b, then:

(1) $$v\left(a,b\right)=\overset{on\;a}{\underset{i}{\sum }}\overset{on\;b}{\underset{j}{\sum }}\frac{q_{i}q_{j}e^{2}}{r_{ij}}+\frac{A}{r_{oo}{}^{12}}-\frac{C}{r_{oo}{}^{6}}$$

where *r_oo_* represents the distance between oxygen atoms. The electrostatic attraction is expressed as a coulombic force between two charges, *q_i_* and *q_j_*, with a distance of *r_ij_* between them. The Van der Waals interaction is expressed as a function that takes attraction and repulsion at the same time. Parameter *A* is the repulsive force of *i* and *j*, whereas parameter *C* represents attraction. The parameters *A* and *C* were carefully chosen to generate reasonable structural and energetic results for liquid water. Parameters values are given below: 

A = 582,000 kcal Å^12^ mol^−1^

C = 595 kcal Å^6^ mol^−1^

q_i_ = −0.834e, q_j_ = 0.417e

Energy criterion of −2.25 kcal mol^−1^ was considered to determine the hydrogen bond between water molecules, as this value correlates with the minimum value of the pair-energy distribution of potential [29]. Further details about TWN are provided elsewhere [6,7].

In TWN analysis, it was assumed that the water distribution in the binding site would be similar if they have similar characteristics. In order to explore this, 26 kinases co-crystallized with the same ligand staurosporine (Table S1 of Supplementary Materials) were subjected to MD simulations in the apo state. Selected kinases belonged to seven major groups of kinome [34]. Among the available crystal structures, PDBs were chosen on the basis of high resolution (≤3 Å) and no missing residues. Furthermore, they were supposed to be in the similar condition, due to the same ligand, staurosporine, in their binding site. After performing the simulations, we obtained 100 trajectory files from the MD results for each protein. Using these files, water in the range of 20 Å around the Asp-Phe-Gly (DFG) motif were extracted to analyze the primary water distribution in the binding site. TWN analysis was performed using a computational protocol reported in our previous works [6,7]. Kinases that displayed a similar TWN pattern as that of IRAK4 were selected for further analysis. Subsequently, we determined the shape similarity, which compares the geometric attributes of how well the water molecules and ligands overlap at the same binding site.

### 3.4. Chemical Library Preparation for Virtual Screening

A lead-like library was downloaded from OTAVA chemicals [24]. Salts were removed and molecular properties, including molecular weight, hydrogen bond acceptors, hydrogen bond donors, rotatable bonds, and AlogP were calculated using a Pipeline Pilot protocol (BIOVIA, San Diego, CA, USA). Lipinski’s ‘rule of five’ [25] was applied, and compounds satisfying the following were selected: molecular weight < 500, number of hydrogen bond donors < 5, number of hydrogen bond acceptors < 10, and logP < 5. Functional class fingerprints of maximum diameter 12 (FCFP12) [35] were employed for the structural diversity of the compounds. A diverse compound library of 380 compounds was established by visual selection.

### 3.5. Pharmacophore Model Generation

A common feature pharmacophore protocol of Discovery Studio 2017 R2 (BIOVIA, San Diego, CA, USA) was employed for pharmacophore analysis. Eight highly active compounds were selected from the known IRAK4 inhibitors [23] to generate a reliable pharmacophore model using the HipHop algorithm [36]. Chosen compounds possessed an IC_50_ value of ≤1 nM. The pIC_50_ range was from 9.52 to 11.00, and the fit value was between 1.10 and 4.99. Firstly, molecular docking studies were performed to generate putative binding modes of these compounds. Then, pharmacophore models were developed, considering pharmacophore features such as hydrogen bond acceptors, hydrogen bond donors, aromatic rings, and hydrophobic groups. The maximum pharmacophore value was set to 10, while a default minimum inter-feature distance value of 2.97 Å was used for generating the pharmacophore.

### 3.6. Molecular Docking

Crystal structures of IRAK4, ASK1, ITK, and LYN, with codes 2NRU [37], 3VW6 [21], 1SM2 [38], and 3A4O [39], respectively, were obtained from PDB [26]. Human PDBs were selected, considering the resolution and no missing residues. Subsequently, protein preparation was carried out as described above. Molecular docking studies were performed with the LigandFit module [40] of Discovery Studio 2017 R2 (BIOVIA, San Diego, CA, USA). The ligand molecules were built and optimized using the Prepare Ligand protocol. Partial charges were applied onto the proteins, as well as ligands using the Momany and Rone method of partial charge estimation. Energy was minimized using the CHARMm forcefield. The binding site of each protein was defined on the basis of the co-crystallized ligand. One hundred docked poses were generated for each ligand, and scored using the Ligscore 1, Ligscore 2, Piecewise linear potential 1 (PLP1), Piecewise linear potential 2 (PLP2), Jain, and Potential of mean force (PMF) scoring functions. The binding modes of the ligands were carefully chosen on the basis of protein-ligand interactions.

### 3.7. Synthesis and Characterization

Unless otherwise indicated, all reactions were run under argon gas. The progress of the reactions was monitored by thin layer chromatography (TLC). All products were determined by ^1^H-NMR spectra and ^13^C-NMR spectra. ^1^H and ^13^C spectra were recorded on a BRUKER 300 MHz spectrometer (Billerica, MA, USA). Chemical shifts are reported relative to internal CDCl_3_ (Me_4_Si, δ 0.0) and DMSO-*d*_6_ (δ 2.50 for ^1^H).

#### 3.7.1. *N*^6^-Benzyl-3-nitro-pyridine-2,6-diamine (**3**-**b**)

To a solution of 2-amino-6-chloro-3-nitropyridine (1.0 equiv.) and benzylamine (2.0 equiv.) in DMF was added K_2_CO_3_ (5.0 equiv.). The reaction mixture was stirred at 80 °C for 19 hr. After cooling at room temperature, H_2_O was slowly added to the reaction mixture. The solid was filtered and washed with H_2_O. Yield: 93.8%, ^1^H-NMR (300 MHz, DMSO-*d*_6_) δ 4.59 (s, 2H), 6.01 (d, *J* = 9.2 Hz, 1H), 7.26 (m, 1H), 7.34 (m, 4H), 7.80 (br. s, 1H), 7.96 (d, *J* = 9.2 Hz, 1H), 8.12 (br. s, 1H), 8.39 (br. s, 1H); ^13^C-NMR (75 MHz, DMSO-*d*_6_) δ 43.80, 102.38, 117.50, 126.99, 127.67, 128.37, 134.46, 139.00, 155.78, 160.46.

#### 3.7.2. *N*^6^-Benzyl-pyridine-2,3,6-triaine (**3**-**c**)

A mixture of **3**-**b** (1.0 equiv.) and SnCl_2_·H_2_O (4.9 equiv.) in ethyl acetate/2-propanol (*v*/*v* 9:1) was stirred at 70 °C for 1 h. Then, NaBH_4_ was added, and the reaction mixture was stirred at 70 °C for 2.5 h. After cooling at room temperature, the solution was diluted with EtOAc and neutralized with saturated K_2_CO_3_. The organic solution was washed with water and brine, and dried with Na_2_SO_4_ and concentrated. The residue was obtained and used without further purification in the next reaction.

#### 3.7.3. Benzyl-(1H-imidazo[4,5-b]pyridine-5-yl)-amine (**3**-**d**)

TsOH·H_2_O (0.1 equiv.) was added to a solution of a **3**-**c** (1.0 equiv.) and HC(OEt)_3_ (2 equiv.) in toluene. The reaction mixture was refluxed for 2.5 h. After cooling at room temperature, the solution was diluted with EtOAc. The organic solution was washed with water and brine, and dried with Na_2_SO_4_ and concentrated. The crude material was purified by column chromatography (silica gel, Hexane/EtOAc) to obtain **3**-**d** (31% in 2 steps). ^1^H-NMR (300 MHz, DMSO-*d*_6_) δ 4.51 (d, *J* = 6.0 Hz, 2H), 6.47 (d, *J* = 8.8 Hz, 1H), 7.01 (br. s, 1H), 7.20 (m, 1H), 7.32 (m, 4H), 7.64 (d, *J* = 8.7 Hz, 1H), 7.88 (s, 1H), 12.32 (br. s, 1H); ^13^C-NMR (75 MHz, DMSO-*d*_6_) δ 44.48, 99.52, 104.74, 126.42, 127.19, 128.15, 128.39, 137.71, 140.73, 146.30, 155.99.

#### 3.7.4. 4-(5-Benzylamino-imidazo[4,5-b]pyridine-1-yl)-benzonitrile (**3**)

A mixture of **3**-**d** (1.0 equiv.), 4-iodobenzonitrile (1.0 equiv.), CuI (0.1 equiv.), Cs_2_CO_3_ (2.1 equiv.), and 1,10-phenanthroline (0.2 equiv.) in DMF was heated to 110 °C for 9.5 hr. After cooling at room temperature, the solution was diluted with EtOAc. The organic solution was washed with water and brine, dried with Na_2_SO_4_, and concentrated. The crude material was purified by column chromatography (silica gel, Hexane/EtOAc) to obtain **3** (34%). ^1^H-NMR (300 MHz, DMSO-*d*_6_) δ 4.49 (d, *J* = 5.8 Hz, 2H), 6.61 (d, *J* = 8.8 Hz, 1H), 7.21 (m, 1H), 7.36 (m, 4H), 7.58 (t, *J* = 5.5 Hz, 1H), 7.79 (d, *J* = 8.8 Hz, 1H), 7.98 (d, *J* = 8.5 Hz, 2H), 8.19 (d, *J* = 8.6 Hz, 2H), 8.56 (s, 1H); ^13^C-NMR (75 MHz, DMSO-*d*_6_) δ 44.86, 106.23, 108.37, 118.55, 121.41, 126.51, 127.23, 127.35, 128.20, 129.47, 133.55, 137.79, 139.70, 140.57, 144.58, 156.10.

### 3.8. In Vitro Assay

Enzymatic assays for IRAK4, ASK1, ITK, and LYN were performed by Eurofins Scientific Inc. Korea (Brussels, Belgium). All assays were performed in duplicate, and the average IC_50_ value was reported.

#### 3.8.1. Enzymatic Assay for IRAK4

IRAK4 (h) was incubated with 8 mM MOPS pH 7.0, 0.2 mM EDTA, 0.33 mg/mL myelin basic protein, 10 mM Mg acetate and [γ-^33^P]-ATP (specific activity approx. 500 cpm/pmol, concentration as required). The reaction was initiated by the addition of a MgATP mix. After incubation for 40 min at room temperature, the reaction was stopped by the addition of 3% phosphoric acid solution. A total of 10 μL of the reaction was then spotted onto a P30 filtermat and washed three times for 5 min in 75 mM phosphoric acid, and once in methanol prior to drying and scintillation counting.

#### 3.8.2. Enzymatic Assay for ASK1

ASK1 (h) was incubated with 8 mM MOPS pH 7.0, 0.2 mM EDTA, 0.33 mg/mL myelin basic protein, 10 mM Mg acetate and [γ-^33^P]-ATP (specific activity approx. 500 cpm/pmol, concentration as required). The reaction was initiated by the addition of the MgATP mix. After incubation for 40 min at room temperature, the reaction was stopped by the addition of 3% phosphoric acid solution. A total of 10 μL of the reaction was then spotted onto a P30 filtermat and washed three times for 5 min in 75 mM phosphoric acid, and once in methanol prior to drying and scintillation counting.

#### 3.8.3. Enzymatic Assay for ITK

ITK (h) was incubated with 8 mM MOPS pH 7.0, 0.2 mM EDTA, 0.33 mg/mL myelin basic protein, 10 mM Mg acetate, and [γ-^33^P]-ATP (specific activity approx. 500 cpm/pmol, concentration as required). The reaction was initiated by the addition of the MgATP mix. After incubation for 40 min at room temperature, the reaction was stopped by the addition of 3% phosphoric acid solution. A total of 10 μL of the reaction was then spotted onto a P30 filtermat and washed three times for 5 min in 75 mM phosphoric acid, and once in methanol prior to drying and scintillation counting.

#### 3.8.4. Enzymatic Assay for LYN

LYN (h) was incubated with 50 mM Tris pH 7.5, 0.1 mM EGTA, 0.1 mM Na_3_VO_4_, 0.1% 6-mercaptoethanol, 0.1 mg/mL poly(Glu, Tyr) 4:1, 10 mM Mg acetate, and [γ-^33^P]-ATP (specific activity approx. 500 cpm/pmol, concentration as required). The reaction was initiated by the addition of the MgATP mix. After incubation for 40 min at room temperature, the reaction was stopped by the addition of 3% phosphoric acid solution. A total of 10 μL of the reaction was then spotted onto a Filtermat A and washed three times for 5 min in 75 mM phosphoric acid, and once in methanol prior to drying and scintillation counting.

## 4. Conclusions

We developed an IRAK4 inhibitor that could be used in the treatment of inflammatory diseases and cancers. We performed simulations and TWN analysis on several kinases to address the critical selectivity issue of kinase inhibitors. Our results suggested that TWN analysis is capable of complementing the limitations of conventional molecular modeling techniques which could not explain the small structural differences. TWNs can aid in identifying selective kinase inhibitors. We obtained a hit molecule for IRAK4 using TWN analysis. Pharmacophore-based virtual screening of commercial chemical library, along with molecular docking studies, provided another hit molecule. Pharmacophore hybridization strategy was applied on the hit molecules to design a novel molecule that significantly inhibited IRAK4 in the in vitro assay. Furthermore, the drug-like physicochemical properties of the designed molecule make it a potential candidate for further evaluation as an IRAK4 inhibitor. Additionally, more effective and selective IRAK4 inhibitors can be developed through further optimization of this molecule.

## Figures and Tables

**Table 1:** Percentage of water networks in various regions (A–E) for IRAK4, ASK1, LYN, and ITK.

Kinase	A (%)	B (%)	C (%)	D (%)	E (%)
IRAK4	25.0	7.1	17.9	35.7	14.3
ASK1	18.5	0.0	29.6	33.3	18.5
LYN	6.3	3.1	9.4	31.3	50.0
ITK	36.0	6.0	16.0	26.0	16.0

**Table 2:** Overall protein sequence and binding site similarities of IRAK4, ASK1, LYN, and ITK.

Total Sequence Similarity (%)/Binding Site Similarity (%)				
Kinase	IRAK4	ASK1	LYN	ITK
IRAK4	100.0/100.0	44.0/40.6	47.9/35.6	44.6/39.6
ASK1	44.0/40.6	100.0/100.0	45.8/46.8	46.8/49.5
LYN	47.9/35.6	45.8/46.8	100.0/100.0	64.0/42.7
ITK	44.6/39.6	46.8/49.5	64.0/42.7	100.0/100.0

**Table 3:** Shape similarity between TWNs and compound **1**.

Kinase	Full Ligand (%)	Around D-Site (%)
IRAK4	22.7	67.6
ASK1	24.2	60.7
LYN	18.2	49.2
ITK	17.4	41.5

## Supplementary Materials

Supplementary materials are available online.

## References

[1] Stout T., Foster P., Matthews D. (2004). High-throughput structural biology in drug discovery: Protein kinases. Curr. Pharm. Des..

[2] Cohen P. (2002). Protein kinases—The major drug targets of the twenty-first century?. Nat. Rev. Drug Discov..

[3] Fernández A., Scheraga H.A. (2003). Insufficiently dehydrated hydrogen bonds as determinants of protein interactions. Proc. Natl. Acad. Sci. USA.

[4] Young T., Abel R., Kim B., Berne B.J., Friesner R.A. (2007). Motifs for molecular recognition exploiting hydrophobic enclosure in protein–ligand binding. Proc. Natl. Acad. Sci. USA.

[5] Murata K., Nagata N., Nakanishi I., Kitaura K. (2010). Ligand shape emerges in solvent dipole ordering region at ligand binding site of protein. J. Comput. Chem..

[6] Jang W.D., Lee M.H., Kang N.S. (2016). Quantitative assessment of kinase selectivity based the water-ring network in protein binding sites using molecular dynamics simulations. J. Mol. Liq..

[7] Jang W.D., Kim J.-T., Kang N.S. (2014). The analysis of water network for kinase selectivity based on the MD simulations. J. Mol. Liq..

[8] Hynes Jr J., Nair S.K., Weinstein D.S., Desai M.C. (2014). Advances in the discovery of small-molecule IRAK4 inhibitors. Annual Reports in Medicinal Chemistry.

[9] Gosu V., Choi S. (2014). Structural dynamic analysis of apo and ATP-bound IRAK4 kinase. Sci. Rep..

[10] Wang Z., Wesche H., Stevens T., Walker N., Yeh W.-C. (2009). IRAK-4 inhibitors for inflammation. Curr. Top. Med. Chem..

[11] Kuglstatter A., Villaseñor A.G., Shaw D., Lee S.W., Tsing S., Niu L., Song K.W., Barnett J.W., Browner M.F. (2007). Cutting edge: IL-1 receptor-associated kinase 4 structures reveal novel features and multiple conformations. J. Immunol..

[12] Ferrao R., Zhou H., Shan Y., Liu Q., Li Q., Shaw D.E., Li X., Wu H. (2014). IRAK4 dimerization and trans-autophosphorylation are induced by Myddosome assembly. Mol. Cell.

[13] Lim J., Altman M.D., Baker J., Brubaker J.D., Chen H., Chen Y., Fischmann T., Gibeau C., Kleinschek M.A., Leccese E. (2015). Discovery of 5-Amino-*N*-(1H-pyrazol-4-yl) pyrazolo [1, 5-a] pyrimidine-3-carboxamide inhibitors of IRAK4. ACS Med. Chem. Lett..

[14] McElroy W.T., Seganish W.M., Herr R.J., Harding J., Yang J., Yet L., Komanduri V., Prakash K.C., Lavey B., Tulshian D. (2015). Discovery and hit-to-lead optimization of 2, 6-diaminopyrimidine inhibitors of interleukin-1 receptor-associated kinase 4. Bioorg. Med. Chem. Lett..

[15] McElroy W.T., Tan Z., Ho G., Paliwal S., Li G., Seganish W.M., Tulshian D., Tata J., Fischmann T.O., Sondey C. (2015). Potent and selective amidopyrazole inhibitors of IRAK4 that are efficacious in a rodent model of inflammation. ACS Med. Chem. Lett..

[16] Seganish W.M., Fischmann T.O., Sherborne B., Matasi J., Lavey B., McElroy W.T., Tulshian D., Tata J., Sondey C., Garlisi C.G. (2015). Discovery and structure enabled synthesis of 2, 6-diaminopyrimidin-4-one IRAK4 inhibitors. ACS Med. Chem. Lett..

[17] Hanisak J., Seganish W.M., McElroy W.T., Tang H., Zhang R., Tsui H.-C., Fischmann T., Tulshian D., Tata J., Sondey C. (2016). Efforts towards the optimization of a bi-aryl class of potent IRAK4 inhibitors. Bioorg. Med. Chem. Lett..

[18] Montesinos-Rongen M., Godlewska E., Brunn A., Wiestler O.D., Siebert R., Deckert M. (2011). Activating L265P mutations of the MYD88 gene are common in primary central nervous system lymphoma. Acta Neuropathol..

[19] Ngo V.N., Young R.M., Schmitz R., Jhavar S., Xiao W., Lim K.-H., Kohlhammer H., Xu W., Yang Y., Zhao H. (2011). Oncogenically active MYD88 mutations in human lymphoma. Nature.

[20] Karaman M.W., Herrgard S., Treiber D.K., Gallant P., Atteridge C.E., Campbell B.T., Chan K.W., Ciceri P., Davis M.I., Edeen P.T. (2008). A quantitative analysis of kinase inhibitor selectivity. Nat. Biotechnol..

[21] Terao Y., Suzuki H., Yoshikawa M., Yashiro H., Takekawa S., Fujitani Y., Okada K., Inoue Y., Yamamoto Y., Nakagawa H. (2012). Design and biological evaluation of imidazo [1, 2-a] pyridines as novel and potent ASK1 inhibitors. Bioorg. Med. Chem. Lett..

[22] Kinnings S.L., Jackson R.M. (2009). Binding site similarity analysis for the functional classification of the protein kinase family. J. Chem. Inf. Model..

[23] Chaudhary D., Robinson S., Romero D.L. (2014). Recent advances in the discovery of small molecule inhibitors of interleukin-1 receptor-associated kinase 4 (IRAK4) as a therapeutic target for inflammation and oncology disorders: Miniperspective. J. Med. Chem..

[24] OTAVA chemicals. www.otavachemicals.com.

[25] Lipinski C.A., Lombardo F., Dominy B.W., Feeney P.J. (1997). Experimental and computational approaches to estimate solubility and permeability in drug discovery and development settings. Adv. Drug Deliv. Rev..

[26] Berman H., Westbrook J., Feng Z., Gilliland G., Bhat T., Weissig H., Shindyalov I., Bourne P. (2000). The protein data bank. Nucleic Acids Res..

[27] Pronk S., Páll S., Schulz R., Larsson P., Bjelkmar P., Apostolov R., Shirts M.R., Smith J.C., Kasson P.M., Van Der Spoel D. (2013). GROMACS 4.5: A high-throughput and highly parallel open source molecular simulation toolkit. Bioinformatics.

[28] Bjelkmar P., Larsson P., Cuendet M.A., Hess B., Lindahl E. (2010). Implementation of the CHARMM force field in GROMACS: Analysis of protein stability effects from correction maps, virtual interaction sites, and water models. J. Chem. Theory Comput..

[29] Jorgensen W.L., Chandrasekhar J., Madura J.D., Impey R.W., Klein M.L. (1983). Comparison of simple potential functions for simulating liquid water. J. Chem. Phys..

[30] Berendsen H.J.C., Postma J.P.M., van Gunsteren W.F., DiNola A., Haak J.R. (1984). Molecular dynamics with coupling to an external bath. J. Chem. Phys..

[31] Nosé S., Klein M. (1983). Constant pressure molecular dynamics for molecular systems. Mol. Phys..

[32] Darden T., York D., Pedersen L. (1993). Particle mesh Ewald: An *N*⋅log(*N*) method for Ewald sums in large systems. J. Chem. Phys..

[33] Hess B., Bekker H., Berendsen H.J., Fraaije J.G. (1997). LINCS: A linear constraint solver for molecular simulations. J. Comput. Chem..

[34] Manning G., Whyte D.B., Martinez R., Hunter T., Sudarsanam S. (2002). The protein kinase complement of the human genome. Science.

[35] Todeschini R., Consonni V. (2009). Molecular Descriptors for Chemoinformatics.

[36] Guner O.F. (2000). Pharmacophore Perception, Development, and Use in Drug Design, Illustrated ed..

[37] Wang Z., Liu J., Sudom A., Ayres M., Li S., Wesche H., Powers J.P., Walker N.P. (2006). Crystal structures of IRAK-4 kinase in complex with inhibitors: A serine/threonine kinase with tyrosine as a gatekeeper. Structure.

[38] Brown K., Long J.M., Vial S.C., Dedi N., Dunster N.J., Renwick S.B., Tanner A.J., Frantz J.D., Fleming M.A., Cheetham G.M. (2004). Crystal structures of interleukin-2 tyrosine kinase and their implications for the design of selective inhibitors. J. Biol. Chem..

[39] Miyano N., Kinoshita T., Nakai R., Kirii Y., Yokota K., Tada T. (2009). Structural basis for the inhibitor recognition of human Lyn kinase domain. Bioorg. Med. Chem. Lett..

[40] Venkatachalam C.M., Jiang X., Oldfield T., Waldman M. (2003). LigandFit: A novel method for the shape-directed rapid docking of ligands to protein active sites. J. Mol. Graph. Model..

