# Supplementary material for: Design of a Novel and Selective IRAK4 Inhibitor Using Topological Water Network Analysis and Molecular Modeling Approaches
Source: Molecules. 2018 Nov 29;23(12):3136. doi: 10.3390/molecules23123136 (PMC6321621; doi:10.3390/molecules23123136)
Supplement: Supplementary file 1 [file molecules-23-03136-s001.pdf]

Supporting Information

Design of a Novel and Selective IRAK4 Inhibitor using Topological Water Network Analysis and Molecular Modeling Approaches

Myeong Hwi Lee [1], Anand Balupuri [1], Ye-rim Jung [1], Sungwook Choi [1], Areum Lee [2], Young Sik Cho [2] and Nam Sook Kang [1,*]

[1] Graduate School of New Drug Discovery and Development, Chungnam National University, 99 Daehak-ro, Yuseong-gu, Daejeon 34134, Korea

[2] College of Pharmacy, Keimyung University, 1095 Dalgubeol-daero, Dalseo-Gu, Daegu 42601, Korea

Table S1. Summary of the simulated protein kinase systems.

Protein	PDB Code	Resolution	Chain	Simulation box size	Water molecules	Contour ions		Total atoms
						Na	Cl	
CDK2	4ERW	2.00	A	8.70 nm × 8.70 nm × 8.70 nm	20335	0	4	65857
ASK1	4BF2	2.11	A	8.24 nm × 8.24 nm × 8.24 nm	17149	6	0	55705
IRAK4	2NRY	2.15	A	8.84 nm × 8.84 nm × 8.84 nm	21406	13	0	68855
GSK3β	1Q3D	2.20	A	9.33 nm × 9.33 nm × 9.33 nm	25164	0	7	81051
DAPK1	1WVY	2.80	A	8.52 nm × 8.52 nm × 8.52 nm	19101	9	0	61750
ITK	1SM2	2.30	A	8.17 nm × 8.17 nm × 8.17 nm	16755	12	0	54435
PDPK1	1OKY	2.30	A	9.08 nm × 9.08 nm × 9.08 nm	23317	0	3	74688
FES	3CBL	1.75	A	8.17 nm × 8.17 nm × 8.17 nm	16730	0	2	54497
LCK	1QPD	2.00	A	8.56 nm × 8.56 nm × 8.56 nm	19432	7	0	62691
ZAP-70	1U59	2.30	A	8.72 nm × 8.72 nm × 8.72 nm	20493	0	6	65999
FYN	2DQ7	2.80	X	8.63 nm × 8.63 nm × 8.63 nm	19941	2	0	64073
LIMK1	3S95	1.65	A	9.34 nm × 9.34 nm × 9.34 nm	25327	0	7	81046
PRKACA	4C34	1.78	A	8.50 nm × 8.50 nm × 8.50 nm	18781	0	8	61438
STK16	2BUJ	2.60	A	9.87 nm × 9.87 nm × 9.87 nm	30344	7	0	95752
STK24	3CKX	2.70	A	8.20 nm × 8.20 nm × 8.20 nm	16784	5	0	54874
PRKCQ	1XJD	2.00	A	8.89 nm × 8.89 nm × 8.89 nm	21733	9	0	69706
LYN	3A4O	3.00	X	8.33 nm × 8.33 nm × 8.33 nm	17731	5	0	57507
CHK1	1NVR	1.80	A	9.20 nm × 9.20 nm × 9.20 nm	24391	5	0	77571
TAO2	2GCD	1.95	A	8.21 nm × 8.21 nm × 8.21 nm	16822	2	0	55428
RPS6KB1	3A62	2.35	A	8.67 nm × 8.67 nm × 8.67 nm	20085	0	9	64916
MAPKAPK2	1NXK	2.70	A	9.34 nm × 9.34 nm × 9.34 nm	25419	0	3	81108
ALK	3LCS	1.95	A	8.68 nm × 8.68 nm × 8.68 nm	20070	4	0	64975
CSK	1BYG	2.40	A	8.42 nm × 8.42 nm × 8.42 nm	18374	0	0	59274
PIM1	1YHS	2.15	A	8.19 nm × 8.19 nm × 8.19 nm	16806	13	0	54789
SRC	3D7T	2.90	B	8.69 nm × 8.69 nm × 8.69 nm	20332	6	0	65429
SYK	1XBC	2.00	A	8.72 nm × 8.72 nm × 8.72 nm	20561	0	1	66125

Table S2. Percentage of water networks in various regions (A–E) for the simulated kinases.

Protein	PDB (%)	A (%)	B (%)	C (%)	D (%)	E (%)
CDK2	4ERW	21.3	6.4	36.2	25.5	10.6
ASK1	4BF2	18.5	0.0	29.6	33.3	18.5
IRAK4	2NRY	25.0	7.1	17.9	35.7	14.3
GSK3β	1Q3D	22.7	2.3	52.3	13.6	9.1
DAPK1	1WVY	28.9	13.2	31.6	7.9	18.4
ITK	1SM2	36.0	6.0	16.0	26.0	16.0
PDPK1	1OKY	19.6	15.2	26.1	15.2	23.9
FES	3CBL	33.3	6.1	18.2	24.2	18.2
LCK	1QPD	24.3	5.4	37.8	13.5	18.9
ZAP-70	1U59	29.7	0.0	24.3	5.4	40.5
FYN	2DQ7	21.4	12.5	35.7	16.1	14.3
LIMK1	3S95	15.8	12.3	26.3	24.6	21.1
PRKACA	4C34	25.9	11.1	29.6	18.5	14.8
STK16	2BUJ	15.5	8.6	37.9	24.1	13.8
STK24	3CKX	29.5	6.8	20.5	11.4	31.8
PRKCQ	1XJD	1.7	15.5	31.0	20.7	31.0
LYN	3A4O	6.3	3.1	9.4	31.3	50.0
CHK1	1NVR	12.5	4.2	35.4	22.9	25.0
TAO2	2GSD	23.5	5.9	17.6	11.8	41.2
RPS6KB1	3A62	6.5	17.4	34.8	8.7	32.6
MAPKAPK2	1NXK	30.0	6.7	33.3	16.7	13.3
ALK	3LCS	30.9	12.7	16.4	16.4	23.6
CSK	1BYG	25.0	17.3	23.1	15.4	19.2
PIM1	1YHS	12.8	35.9	12.8	12.8	25.6
SRC	3D7T	23.3	10.0	28.3	18.3	20.0
SYK	1XBC	12.2	2.0	44.9	18.4	22.4

Table S3. (A) Sequence similarity of the D-site among the simulated kinases.

Kinase	CDK2	ASK1	IRAK4	GSK3β	DAPK1	ITK	PDPK1	FES	LCK	ZAP-70	FYN	LIMK1	PRKACA
**CDK2**	100	20	40	40	40	40	40	60	60	0	60	40	40
**ASK1**	20	100	80	60	60	40	20	60	60	40	60	80	80
**IRAK4**	40	80	100	60	40	60	40	40	80	20	80	60	60
**GSK3β**	40	60	60	100	40	40	20	40	60	20	60	60	60
**DAPK1**	40	60	40	40	100	40	60	60	40	60	40	60	80
**ITK**	40	40	60	40	40	100	60	60	80	20	60	60	40
**PDPK1**	40	20	40	20	60	60	100	40	60	40	40	40	40
**FES**	60	60	40	40	60	60	40	100	60	40	60	80	60
**LCK**	60	60	80	60	40	80	60	60	100	20	80	80	40
**ZAP-70**	0	40	20	20	60	20	40	40	20	100	20	40	40
**FYN**	60	60	80	60	40	60	40	60	80	20	100	80	40
**LIMK1**	40	80	60	60	60	60	40	80	80	40	80	100	60
**PRKACA**	40	80	60	60	80	40	40	60	40	40	40	60	100
**STK16**	20	40	40	40	20	40	40	40	60	20	60	60	20
**STK24**	20	80	60	40	60	40	20	60	60	60	60	80	60
**PRKCQ**	60	60	60	20	60	60	40	80	60	40	60	60	60
**LYN**	40	60	60	60	60	60	60	60	80	40	80	80	40
**CHK1**	20	40	40	20	80	20	60	40	40	60	40	40	60
**TAO2**	25	60	60	40	40	40	20	40	60	20	60	60	40
**RPS6KB1**	40	60	60	40	100	40	60	60	60	60	60	60	80
**MAPKAPK2**	40	60	60	40	80	40	60	80	60	40	60	60	80
**ALK**	40	60	40	20	80	40	40	80	40	60	40	60	60
**CSK**	40	60	60	60	60	60	60	60	80	40	80	80	40
**PIM1**	50	17	33	50	33	33	33	50	50	17	33	33	33
**SRC**	40	60	60	60	60	60	60	60	80	40	80	80	40
**SYK**	20	40	40	40	40	40	60	40	40	60	40	40	40

(B) Sequence similarity of the D-site among the simulated kinases.

Kinase	STK16	STK24	PRKCQ	LYN	CHK1	TAO2	RPS6KB1	MAPKAPK2	ALK	CSK	PIM1	SRC	SYK
**CDK2**	20	20	60	40	20	25	40	40	40	40	50	40	20
**ASK1**	40	80	60	60	40	60	60	60	60	60	17	60	40
**IRAK4**	40	60	60	60	40	60	60	60	40	60	33	60	40
**GSK3β**	40	40	20	60	20	40	40	40	20	60	50	60	40
**DAPK1**	20	60	60	60	80	40	100	80	80	60	33	60	40
**ITK**	40	40	60	60	20	40	40	40	40	60	33	60	40
**PDPK1**	40	20	40	60	60	20	60	60	40	60	33	60	60
**FES**	40	60	80	60	40	40	60	80	80	60	50	60	40
**LCK**	60	60	60	80	40	60	60	60	40	80	50	80	40
**ZAP-70**	20	60	40	40	60	20	60	40	60	40	17	40	60
**FYN**	60	60	60	80	40	60	60	60	40	80	33	80	40
**LIMK1**	60	80	60	80	40	60	60	60	60	80	33	80	40
**PRKACA**	20	60	60	40	60	40	80	80	60	40	33	40	40
**STK16**	100	40	20	60	20	40	20	20	20	60	17	60	40
**STK24**	40	100	60	60	40	60	60	60	60	60	17	60	40
**PRKCQ**	20	60	100	40	60	40	80	80	80	40	33	40	40
**LYN**	60	60	40	100	40	60	60	40	60	100	30	100	60
**CHK1**	20	40	60	40	100	40	80	60	60	40	17	40	40
**TAO2**	40	60	40	60	40	100	40	40	40	60	17	60	40
**RPS6KB1**	20	60	80	60	80	40	100	80	80	60	33	60	40
**MAPKAPK2**	20	60	80	40	60	40	80	100	60	40	33	40	40
**ALK**	20	60	80	60	60	40	80	60	100	60	33	60	40
**CSK**	60	60	40	100	40	60	60	40	60	100	33	100	60
**PIM1**	17	17	33	30	17	17	33	33	33	33	100	33	33
**SRC**	60	60	40	100	40	60	60	40	60	100	33	100	60
**SYK**	40	40	40	60	40	40	40	40	40	60	33	60	100

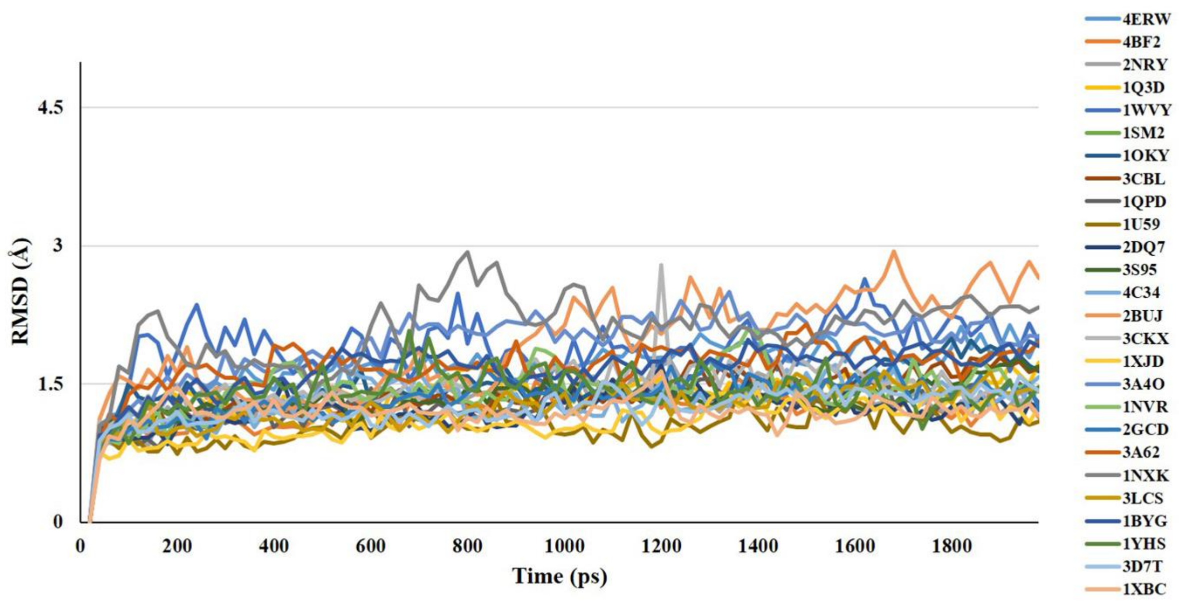

Figure S1. RMSD curves of the simulated kinases during 2000 ps MD simulation.

Shape similarity codes

```
import java.io.*;
import java.util.*;

import javax.vecmath.Point3d;

/*
 * Shape Similarity
 * Input files:
 * Query Structure : .sd files
 * Target Structures : Saved .sd files in DS Generate Conformations
 *
 */

public class ShapeSim_Lig_Wat {

    static String QueryFileName = "STU";
    static String TargetFileName = "Wat";

    static int qatom;
    static int tatom;

    public static void main(String[] args) throws IOException {

        File dir = new File("E:₩₩cheminfo₩₩ShapeSim_Lig_Wat");

        if(!dir.exists() || !dir.isDirectory()) {
            System.out.println("Not a validated directory");
            System.exit(0);
        }

        File nf = new File("E:₩₩cheminfo₩₩ShapeSim_Lig_Wat₩₩results₩₩");
        if(!nf.exists()) {
            nf.mkdirs();
        }
    /*
        else {
            System.out.println("Remove Results Folder!");
            System.exit(0);
        }
    */
            String refName = QueryFileName;

            FileReader fr = new FileReader("E:₩₩cheminfo₩₩ShapeSim_Lig_Wat₩₩"+refName+".sd");    //
Ligand file

            Scanner sr = new Scanner(fr);

            while(sr.hasNextLine()) {
                String line = sr.nextLine();

                if(line.contains("V2000")) {
                    StringTokenizer st = new StringTokenizer(line);
                    qatom = Integer.parseInt(st.nextToken());
    //                  System.out.println(refName+" atom # is "+qatom);
                    break;
                }
            }
            fr.close();
```

```
			fr = new FileReader("E:\\cheminfo\\ShapeSim_Lig_Wat\\"+refName+".sd");

			double[] qx = new double[qatom*3];
			double[] qy = new double[qatom*3];
			double[] qz = new double[qatom*3];

			Scanner sr2 = new Scanner(fr);

			while(sr2.hasNextLine()) {
				String line = sr2.nextLine();

				if(line.contains("V2000")) {
					for(int qi=0; qi<qatom; qi++) {
						line = sr2.nextLine();
						StringTokenizer qt = new StringTokenizer(line);
						qx[qi] = Double.parseDouble(qt.nextToken());
						qy[qi] = Double.parseDouble(qt.nextToken());
						qz[qi] = Double.parseDouble(qt.nextToken());
					}
					break;
				}
			} // end while

				FileReader fr2 = new FileReader("E:\\cheminfo\\ShapeSim_Lig_Wat
\\"+TargetFileName+".sd");	// Wat

				Scanner sr_cnt = new Scanner(fr2);

				String line_cnt = "";
				int wat_cnt = 0;
				while(sr_cnt.hasNextLine()) {
					line_cnt = sr_cnt.nextLine();
					if(line_cnt.contains("V2000")) {
						wat_cnt++;
					}
				}
				tatom = wat_cnt;

				sr_cnt.close();
				fr2.close();

				FileWriter fw = new FileWriter("E:\\cheminfo\\ShapeSim_Lig_Wat\\results
\\"+refName+"_"+TargetFileName+".out");	// results file
				fr2 = new FileReader("E:\\cheminfo\\ShapeSim_Lig_Wat
\\"+TargetFileName+".sd");	// Wat
		//		BufferedReader sr3 = new BufferedReader(fr2);
				Scanner sr3 = new Scanner(fr2);

				String line = "";

				double[] tx = new double[tatom*3];
				double[] ty = new double[tatom*3];
				double[] tz = new double[tatom*3];

				int ti = 0;

				while(sr3.hasNextLine()) {
					line = sr3.nextLine();
```

```
							if(line.contains("V2000")) {

								line = sr3.nextLine();


									StringTokenizer st2 = new StringTokenizer(line);
									tx[ti] = Double.parseDouble(st2.nextToken());
									ty[ti] = Double.parseDouble(st2.nextToken());
									tz[ti] = Double.parseDouble(st2.nextToken());

									ti++;

							}
						}

								double[] moment1 = generateMoments(qatom, qx, qy, qz);
								double[] moment2 = generateMoments(tatom, tx, ty, tz);
								double sum = 0;
								for (int si=0; si<moment1.length; si++) {
									sum += Math.abs(moment1[si] - moment2[si]);
								}
								double sim = (1.0 / (1.0 + sum/12.0));

								fw.write(QueryFileName+"\t"+sim+"\n");
								System.out.println(sim);

						sr3.close();
						fr2.close();
						fw.close();
	} // end main

	private static Point3d getGeometricCenter(int atomNum, double[] x, double[] y, double[] z) {

		double xi = 0;
		double yi = 0;
		double zi = 0;

		for(int i=0; i<x.length; i++) {
			xi += x[i];
			yi += y[i];
			zi += z[i];
		}
		xi /= atomNum;
		yi /= atomNum;
		zi /= atomNum;

		return new Point3d(xi,yi,zi);
	}

private static float mu1(double[] x) {
    float sum = 0;
    for (double aX : x) {
        sum += aX;
    }
    return sum / x.length;
}
```

```java
private static float mu2(double[] x, double mean) {
    float sum = 0;
    for (double aX : x) {
        sum += (aX - mean) * (aX - mean);
    }
    return sum / (x.length - 1);
}

private static float mu3(double[] x, double mean, double sigma) {
    float sum = 0;
    for (double aX : x) {
        sum += ((aX - mean) / sigma) * ((aX - mean) / sigma) * ((aX - mean) / sigma);
    }
    return sum / x.length;
}


public static double[] generateMoments(int atomNum, double[] x, double[] y, double[] z) {

//    System.out.println(atomNum);

      Point3d ctd = getGeometricCenter(atomNum, x, y, z);
      Point3d cst = new Point3d();
      Point3d fct = new Point3d();
      Point3d ftf = new Point3d();

      double[] distCtd = new double[atomNum];
      double[] distCst = new double[atomNum];
      double[] distFct = new double[atomNum];
      double[] distFtf = new double[atomNum];

      int counter = 0;
      double min = Double.MAX_VALUE;
      double max = Double.MIN_VALUE;

      //dist to ctd
      for(int i=0; i<atomNum; i++) {
            Point3d p = new Point3d(x[i], y[i], z[i]);
            double d = p.distance(ctd);
            distCtd[counter++] = d;

            if(d < min) {
                  cst.x = p.x;
                  cst.y = p.y;
                  cst.z = p.z;
                  min = d;
            }
            if(d > max) {
                  fct.x = p.x;
                  fct.y = p.y;
                  fct.z = p.z;
                  max = d;
            }
      }

      //dist to cst
      counter = 0;
      for(int i=0; i<atomNum; i++) {
            Point3d p = new Point3d(x[i], y[i], z[i]);
```

```
                double d = p.distance(cst);
                distCst[counter++] = d;
        }

        //dist to fct
        counter = 0;
        max = Double.MIN_VALUE;
        for(int i=0; i<atomNum; i++) {
                Point3d p = new Point3d(x[i], y[i], z[i]);
                double d = p.distance(fct);
                distFct[counter++] = d;

                if (d > max) {
                        ftf.x = p.x;
                        ftf.y = p.y;
                        ftf.z = p.z;
                        max = d;
                }
        }

        //dist to ftf
        counter = 0;
        for(int i=0; i<atomNum; i++) {
                Point3d p = new Point3d(x[i], y[i], z[i]);
                double d = p.distance(ftf);
                distFtf[counter++] = d;
        }

        double[] moments = new double[12];

        double mean = mu1(distCtd);
        double sigma2 = mu2(distCtd, mean);
        double skewness = mu3(distCtd, mean, Math.sqrt(sigma2));
        moments[0] = mean;
        moments[1] = sigma2;
        moments[2] = skewness;

        mean = mu1(distCst);
      sigma2 = mu2(distCst, mean);
      skewness = mu3(distCst, mean, Math.sqrt(sigma2));
      moments[3] = mean;
      moments[4] = sigma2;
      moments[5] = skewness;


      mean = mu1(distFct);
      sigma2 = mu2(distFct, mean);
      skewness = mu3(distFct, mean, Math.sqrt(sigma2));
      moments[6] = mean;
      moments[7] = sigma2;
      moments[8] = skewness;

      mean = mu1(distFtf);
      sigma2 = mu2(distFtf, mean);
      skewness = mu3(distFtf, mean, Math.sqrt(sigma2));
      moments[9] = mean;
      moments[10] = sigma2;
      moments[11] = skewness;


          return moments;
    }



}
```
